# Cytomegalovirus Retinitis Mimicking Acute Retinal Necrosis During Upadacitinib Therapy

**DOI:** 10.7759/cureus.116160

**Published:** 2026-09-13

**Authors:** Takahiro Matsuoka, Hiroki Kaneko

**Affiliations:** 1 Ophthalmology, Shizuoka Red Cross Hospital, Shizuoka, JPN; 2 Ophthalmology, Hamamatsu University School of Medicine, Hamamatsu, JPN

**Keywords:** acute retinal necrosis, aqueous humor pcr, cytomegalovirus retinitis, immunosuppression, retinal vasculitis, upadacitinib

## Abstract

Cytomegalovirus (CMV) retinitis classically occurs in patients with advanced human immunodeficiency virus infection but may also affect non-HIV patients with impaired cellular immunity and can resemble acute retinal necrosis (ARN). A 74-year-old woman receiving upadacitinib 15 mg/day for rheumatoid arthritis presented with decreased vision and a central dark scotoma in the left eye. Widefield montage imaging obtained with the Mirante SLO/OCT platform (NIDEK Co., Ltd., Gamagori, Aichi, Japan) showed temporal to inferotemporal retinal whitening with hemorrhages, vitreous haze, marked perivascular changes, and occlusive vasculitis. Intravenous acyclovir was started for presumed ARN. Aqueous humor polymerase chain reaction subsequently detected CMV DNA at 6.85 × 10⁴ copies/mL, whereas herpes simplex virus and varicella-zoster virus results were negative; HIV RNA was undetectable and peripheral-blood CMV antigenemia was negative. Treatment was changed to oral valganciclovir, and upadacitinib was discontinued. The valganciclovir dose was reduced after renal dysfunction developed. The retinal whitening regressed into atrophic scars without retinal detachment or fellow-eye involvement, but vision remained poor because of macular ischemia and outer retinal damage. This case illustrates that CMV retinitis in a non-HIV immunosuppressed patient may present with an ARN-like phenotype and severe occlusive vasculitis. Early ocular fluid testing can identify the pathogen and redirect antiviral treatment; the temporal association with upadacitinib should not be interpreted as proof of drug-specific causality.

## Introduction

Cytomegalovirus (CMV) retinitis is most strongly associated with advanced human immunodeficiency virus (HIV) infection, but it also occurs in HIV-negative patients with impaired cellular immunity. Relevant clinical backgrounds include systemic immunosuppressive therapy, malignancy, diabetes mellitus, and advanced age [1-3]. In HIV-negative patients, CMV retinitis may be accompanied by more prominent vitreous inflammation and occlusive vascular changes than the classic phenotype described in acquired immunodeficiency syndrome [1-3].

Acute retinal necrosis (ARN) is a clinical syndrome characterized by peripheral retinal necrosis, rapid progression, occlusive vasculopathy, and intraocular inflammation. These findings are not entirely pathogen-specific, and CMV retinitis can occasionally produce an ARN-like appearance. Because herpes simplex virus (HSV), varicella-zoster virus (VZV), and CMV require different antiviral strategies, ocular-fluid polymerase chain reaction (PCR) is important when the clinical phenotype alone does not establish the causative pathogen [4,5].

Upadacitinib is a selective Janus kinase 1 inhibitor used to treat rheumatoid arthritis and other immune-mediated diseases. CMV retinitis during upadacitinib therapy has been reported previously [6-8]. We describe a non-HIV patient with PCR-confirmed CMV retinitis that presented as ARN-like necrotizing retinitis with extensive occlusive vasculitis. The focus of this report is the diagnostic shift prompted by aqueous humor PCR rather than attribution of causality to a single immunomodulatory drug.

## Case presentation

A 74-year-old woman was referred for suspected retinal necrosis in the left eye. She had rheumatoid arthritis and had received upadacitinib 15 mg/day for approximately three years before presentation. Prednisolone had previously been used for approximately five years and had been discontinued approximately 20 months before presentation. Her medical history also included Hodgkin lymphoma, hypertension, type 2 diabetes mellitus, and bilateral pseudophakia after cataract surgery approximately seven years earlier. Complete historical medication regimens for Hodgkin lymphoma, hypertension, and diabetes, including drug names, doses, and treatment durations, could not be reconstructed from the available records. Her body weight at admission was 39.2 kg.

At the initial ophthalmology visit (Day 0), she reported decreased vision and a central dark scotoma in the left eye. Best-corrected visual acuity was 0.00 logarithm of the minimum angle of resolution (logMAR; 20/20 Snellen equivalent) in the right eye and 0.15 logMAR (approximately 20/28) in the left eye. Intraocular pressure (IOP), measured using an iCare rebound tonometer (iCare Finland Oy, Vantaa, Finland), was 11.7 mmHg in the right eye and 35.5 mmHg in the left eye.

Widefield montage color fundus photography obtained with the Mirante SLO/OCT platform (NIDEK Co., Ltd., Gamagori, Aichi, Japan) showed extensive whitish retinal lesions from the temporal to inferotemporal retina, with scattered hemorrhages, vitreous haze, marked perivascular changes, and occlusive vascular changes (Figure 1A). Optical coherence tomography (OCT) through the fovea showed a relatively preserved foveal contour without prominent cystoid macular edema (Figure 1B).

**Figure 1:**
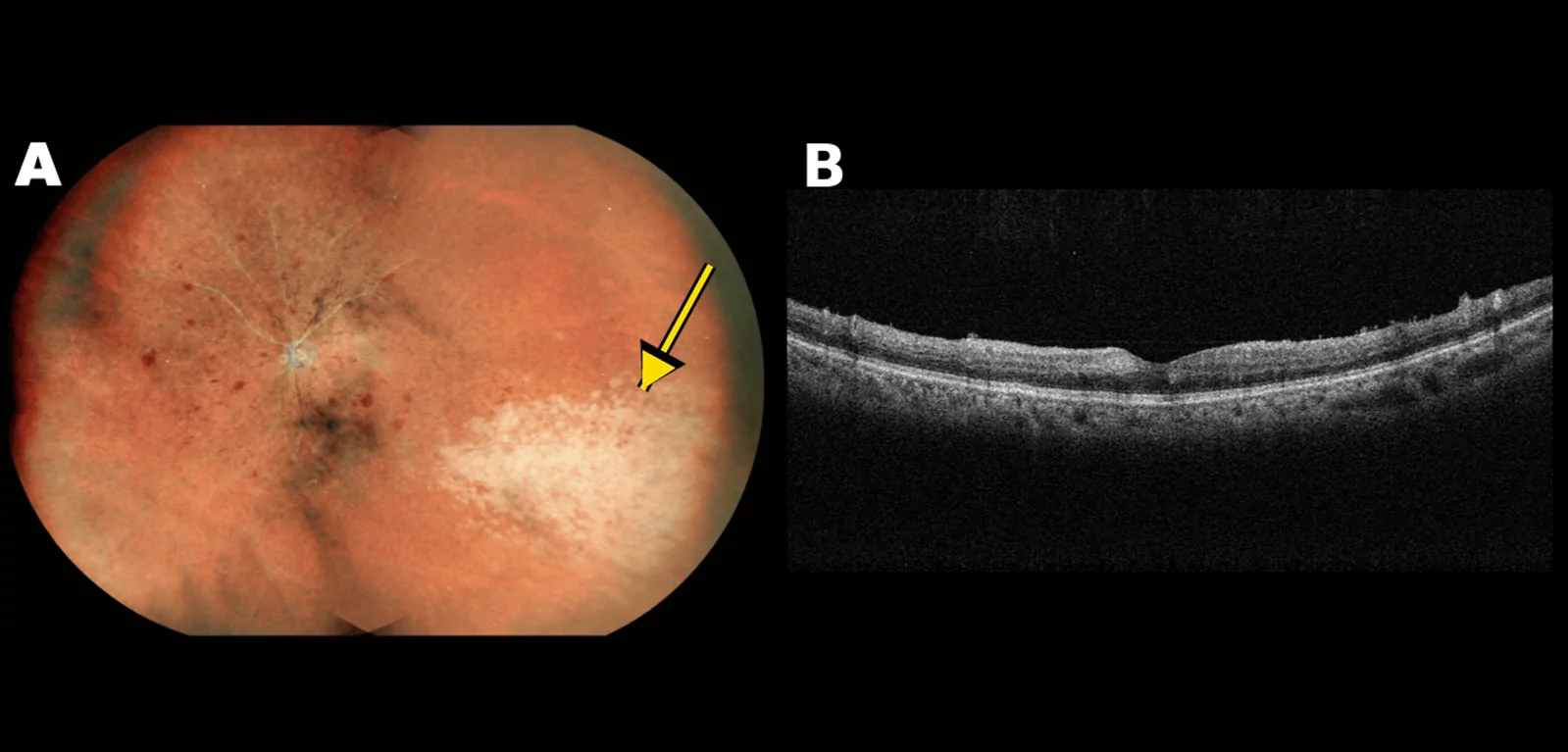
Initial fundus and optical coherence tomography findings in the left eye. (A) Widefield montage color fundus photography at presentation shows extensive temporal to inferotemporal whitish retinal lesions with scattered hemorrhages, vitreous haze, and marked perivascular and occlusive vascular changes. The yellow arrow indicates a representative active lesion. (B) Optical coherence tomography through the fovea shows a relatively preserved foveal contour without prominent cystoid macular edema. Both panels were acquired with the Mirante SLO/OCT platform.

An aqueous humor sample was collected from the left eye on Day 0 by anterior chamber paracentesis using a 30-gauge needle. Approximately 0.1 mL was placed in a sterile tube and transported at 4°C to Kobe City Eye Hospital (Kobe, Japan) for external laboratory testing. Testing comprised a qualitative strip-based PCR panel for ocular pathogens and quantitative PCR for CMV. The sample was collected before intravenous acyclovir was initiated on Day 1 for presumed ARN, based on the peripheral necrotizing retinitis, vitreous haze, and occlusive vasculopathy.

On Day 5, before valganciclovir initiation, serum creatinine was 0.84 mg/dL and the estimated glomerular filtration rate was 50.4 mL/min/1.73 m². Creatinine clearance was retrospectively estimated at 36.4 mL/min using the Cockcroft-Gault equation, the patient’s age of 74 years, and her admission body weight of 39.2 kg. Hemoglobin was 7.4 g/dL, indicating severe pre-existing anemia. The aqueous humor PCR results became available on Day 6. CMV DNA was detected at 6.85 × 10⁴ copies/mL, whereas HSV-1, HSV-2, and VZV PCR results were negative. HIV-1 RNA was undetectable. The diagnosis was revised to CMV retinitis in a non-HIV immunosuppressed patient.

On Day 7, antiviral therapy was changed to oral valganciclovir 900 mg once daily following consultation with the treating hematologist. The medical record documented an individualized treatment decision that prioritized safety in view of reduced renal function, severe anemia, advanced age, and overall clinical condition. No intravenous or intravitreal ganciclovir was administered. Following consultation with rheumatology, the decision to discontinue upadacitinib was made on Day 7, and the final dose was taken on that day.

For elevated IOP in the left eye, topical fixed-combination dorzolamide/timolol was started on Day 7 and continued throughout ophthalmic follow-up. IOP reached 37 mmHg on Day 8. Brimonidine and ripasudil were added on Day 8 and continued until hospital discharge. Peripheral-blood CMV antigenemia testing on a sample collected on Day 8, after CMV-directed treatment had begun, was negative.

Widefield montage fluorescein angiography on Day 14 demonstrated marked leakage from the optic disc and retinal vessels, occlusive retinal vasculitis, and hypofluorescent areas corresponding to the temporal necrotic lesions (Figure 2).

**Figure 2:**
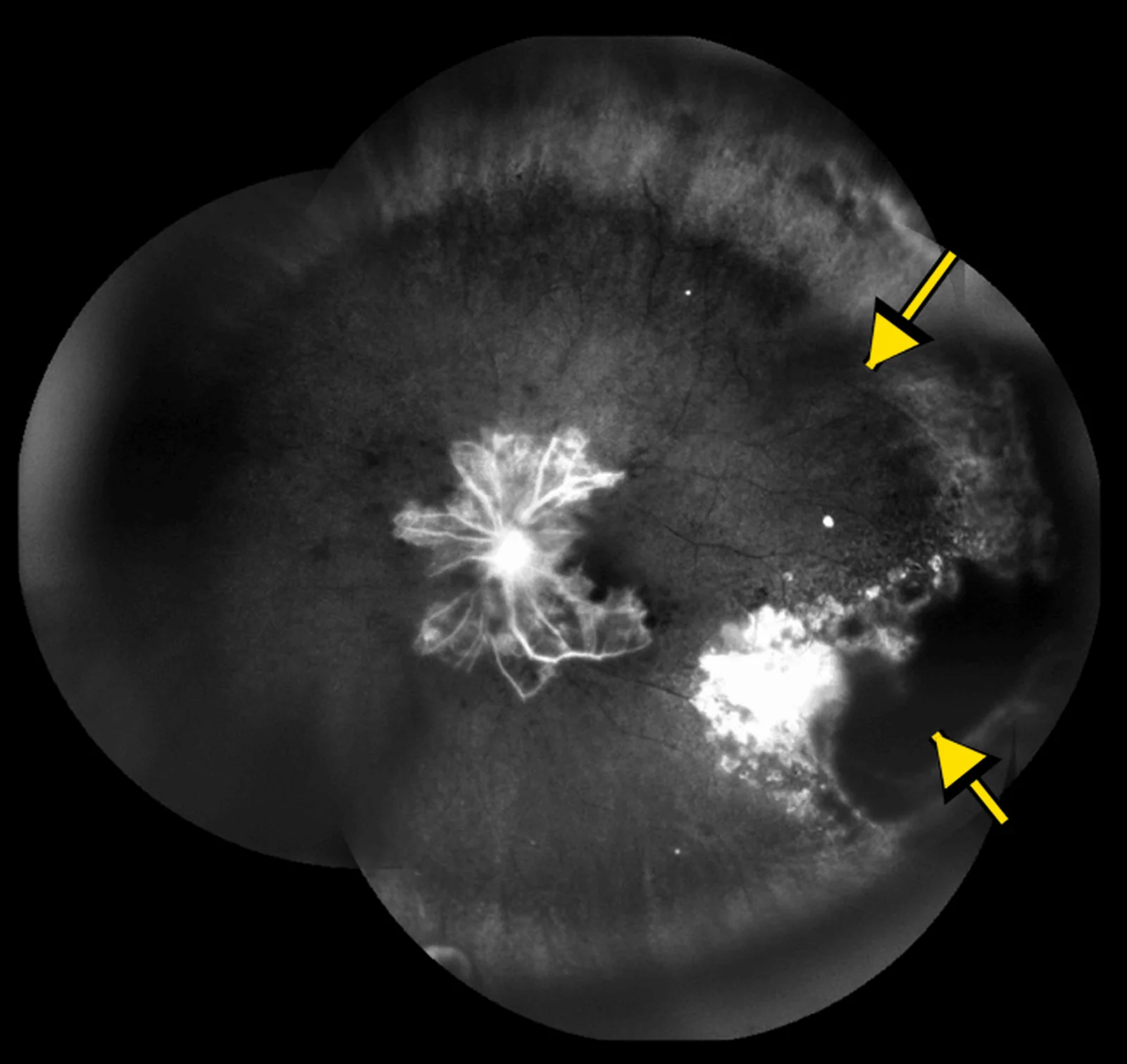
Fluorescein angiography of the left eye on Day 14. Widefield montage fluorescein angiography demonstrates marked leakage from the optic disc and retinal vessels, occlusive vascular changes, and hypofluorescent areas corresponding to the temporal necrotic lesions. The yellow arrows identify representative temporal areas of vascular abnormality and hypofluorescence. The image was acquired with the Mirante SLO/OCT platform.

Valganciclovir was subsequently reduced to 450 mg once daily following consultation with the treating hematologist because of worsening renal function, with the patient’s overall clinical condition also taken into consideration. During hospitalization, the patient developed acute pneumonia requiring systemic corticosteroid therapy for respiratory management. Non-HIV* Pneumocystis jirovecii *pneumonia was considered clinically and radiographically, but *Pneumocystis jirovecii* PCR was negative and the diagnosis was not microbiologically confirmed. Serum creatinine reached 1.61 mg/dL on Day 16 during the intercurrent systemic illness and concomitant treatment that included trimethoprim-sulfamethoxazole.

The reduced valganciclovir dose was continued until approximately 13 weeks after presentation. No further expansion of the retinal whitening was observed after CMV-directed therapy began. Serial widefield montage photographs showed regression of active retinal whitening and evolution into sharply demarcated atrophic scars. At approximately 10 weeks, the lesions appeared inactive without obvious extension (Figure 3).

**Figure 3:**
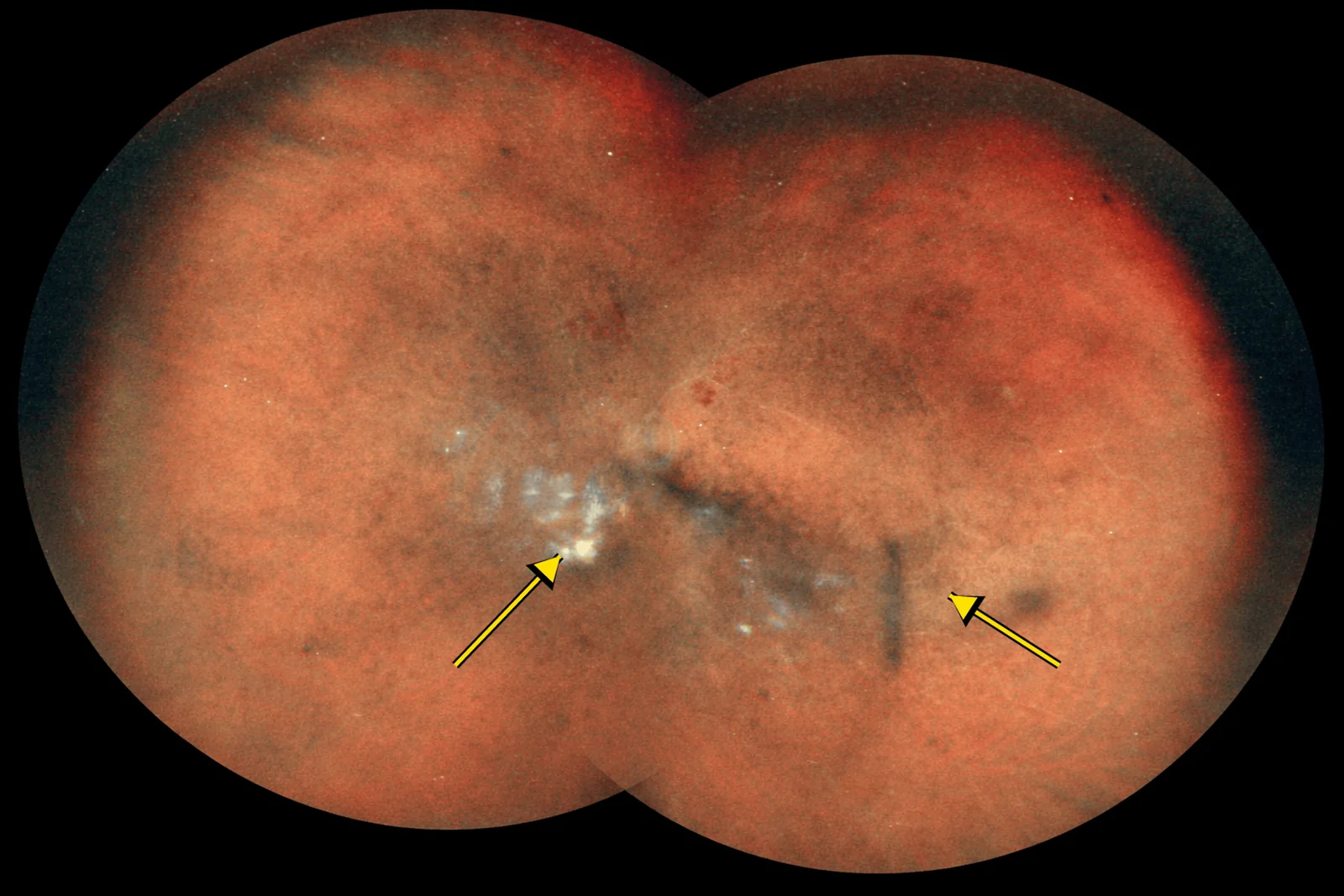
Follow-up fundus photograph of the left eye at approximately 10 weeks. Widefield montage color fundus photography shows regression of active retinal whitening and evolution into inactive atrophic scars without obvious further extension. The yellow arrows indicate representative scars. The image was acquired with the Mirante SLO/OCT platform.

At the final ophthalmic follow-up, approximately 19 weeks after presentation, best-corrected visual acuity was -0.08 logMAR (approximately 20/17) in the right eye and 1.30 logMAR (approximately 20/400) in the left eye. IOP was 13.9 mmHg in the right eye and 21.0 mmHg in the left eye. Macular ischemia and outer retinal damage were considered contributors to the limited visual recovery. No retinal tear, retinal detachment, or fellow-eye involvement occurred. No later ophthalmic follow-up was available. CD4-positive lymphocyte subset measurements and repeat quantitative ocular-fluid PCR were not obtained.

## Discussion

The peripheral retinal whitening, vitreous haze, hemorrhages, and occlusive vasculopathy initially supported a working diagnosis of ARN. However, these findings alone did not establish the causative virus. Detection of CMV DNA in aqueous humor, together with negative HSV and VZV results, redirected treatment from acyclovir to valganciclovir [4,5]. The aqueous sample was obtained before acyclovir initiation, clarifying the temporal relationship between diagnostic sampling and empirical treatment. The negative peripheral-blood CMV antigenemia result did not contradict the ocular PCR diagnosis; however, that blood sample was collected after CMV-directed treatment had begun and should not be interpreted as a pretreatment assessment.

Fluorescein angiography documented the severity of retinal vascular involvement but was not etiologically diagnostic. In this case, imaging and ocular-fluid testing served complementary purposes: imaging characterized the retinal lesions and vascular changes, whereas PCR provided pathogen-specific information. Regression of active retinitis did not result in substantial visual recovery, with macular ischemia and outer retinal damage considered possible contributors. The unilateral IOP elevation may have reflected inflammation-related impairment of aqueous outflow in the affected left eye; however, the precise mechanism could not be established.

Previous reports provide context for CMV retinitis during upadacitinib therapy. Hirai et al. described bilateral CMV retinitis with extensive white retinal lesions and hemorrhages [6]. Park and Lee reported a case combining features of ARN and CMV retinitis, in which aqueous humor PCR established the diagnosis and fluorescein angiography demonstrated extensive nonperfusion [7]. Johnson et al. described subsequent retinal ischemia and vitreous hemorrhage following CMV retinitis during upadacitinib therapy [8]. The present case adds an example of a unilateral ARN-like phenotype with severe occlusive vasculitis and persistent visual impairment despite clinical regression of the retinitis.

These reports describe a temporal association and do not establish that upadacitinib independently causes CMV retinitis. The patient had several potentially relevant clinical factors, including advanced age, diabetes mellitus, a history of Hodgkin lymphoma, and previous corticosteroid exposure. Incomplete information regarding historical lymphoma treatment and the absence of CD4-positive lymphocyte measurements limited assessment of her immune status. Upadacitinib withdrawal and initiation of valganciclovir occurred at approximately the same time, preventing attribution of improvement to either intervention alone.

The initial valganciclovir regimen requires cautious interpretation. Although the documented decision considered renal function and other toxicity risks, 900 mg once daily did not correspond to the renal-function-based induction schedule in the prescribing information. For a creatinine clearance of 25-39 mL/min, that schedule specifies 450 mg once daily for initial treatment [9]. The regimen used in this patient therefore should not be presented as standard renal-adjusted therapy or as a validated safer alternative. Severe pre-existing anemia was an additional concern given the hematologic toxicity warnings for valganciclovir [9].

The subsequent renal impairment occurred during systemic illness and concomitant treatment with trimethoprim-sulfamethoxazole and other medications, so a specific causal relationship with valganciclovir could not be established. Similarly, clinical regression of the retinal lesions without repeat quantitative ocular-fluid PCR does not establish virologic clearance. The nonstandard initial regimen, subsequent dose reduction, and simultaneous changes in immunomodulatory treatment limit conclusions about antiviral efficacy and tolerability. This single case cannot determine an optimal valganciclovir dose or compare systemic treatment alone with treatment that includes intravitreal antiviral therapy.

Additional limitations include the single-patient design, incomplete standardized grading of anterior chamber inflammation and vitreous haze, incomplete historical medication data, and the absence of follow-up beyond approximately 19 weeks. The principal contribution of this report is therefore diagnostic: CMV should remain in the differential diagnosis of ARN-like retinitis in an immunosuppressed non-HIV patient, and early ocular-fluid PCR can help identify the pathogen and guide antiviral selection.

## Conclusions

CMV retinitis can present as ARN-like necrotizing retinitis with severe occlusive vasculitis in a non-HIV patient receiving systemic immunomodulatory therapy. Fundus examination and fluorescein angiography define the phenotype and severity but do not reliably identify the causative virus. Aqueous humor PCR obtained early in the diagnostic evaluation can redirect antiviral treatment when the clinical appearance suggests ARN but the host background also raises concern for CMV. The association with upadacitinib should be regarded as temporal rather than causal, and the individualized antiviral regimen reported here should not be interpreted as evidence of an optimal or safer dosing strategy.

## References

[1] Munro M, Yadavalli T, Fonteh C, Arfeen S, Lobo-Chan AM (2019). Cytomegalovirus retinitis in HIV and non-HIV individuals. Microorganisms.

[2] Downes KM, Tarasewicz D, Weisberg LJ, Cunningham ET Jr (2016). Good syndrome and other causes of cytomegalovirus retinitis in HIV-negative patients-case report and comprehensive review of the literature. J Ophthalmic Inflamm Infect.

[3] Zhang J, Kamoi K, Zong Y, Yang M, Zou Y, Miyagaki M, Ohno-Matsui K (2024). Cytomegalovirus retinitis: clinical manifestations, diagnosis and treatment. Viruses.

[4] Kalogeropoulos D, Afshar F, Kalogeropoulos C, Vartholomatos G, Lotery AJ (2024). Diagnostic and therapeutic challenges in acute retinal necrosis; an update. Eye (Lond).

[5] Miyazaki D, Shimizu D, Shimizu Y (2018). Diagnostic efficacy of real-time PCR for ocular cytomegalovirus infections. Graefes Arch Clin Exp Ophthalmol.

[6] Hirai H, Akai Y, Ogata N, Ueda T (2023). Cytomegalovirus retinitis in a patient taking upadacitinib: a case report. Cureus.

[7] Park SJ, Lee YJ (2026). Cytomegalovirus retinitis during upadacitinib monotherapy for rheumatoid arthritis: a case report. J Rheum Dis.

[8] Johnson CD, El Zein L, Yamanuha J (2026). From infection to infarction: cytomegalovirus retinitis complicated by retinal ischemia and vitreous hemorrhage in the setting of JAK inhibition - a case report. BMC Ophthalmol.

[9] (2026). Tanabe Pharma Corporation. Valixa tablets 450 mg and Valixa dry syrup 5000 mg: Japanese electronic prescribing information (in Japanese). December 2025 revision. https://www.pmda.go.jp/PmdaSearch/bookSearch/01/04987128319565.

